# The Impact of Excluding Trials from Network Meta-Analyses – An Empirical Study

**DOI:** 10.1371/journal.pone.0165889

**Published:** 2016-12-07

**Authors:** Jing Zhang, Yiping Yuan, Haitao Chu

**Affiliations:** 1 Department of Epidemiology and Biostatistics, University of Maryland School of Public Health College Park, Maryland, United States of America; 2 School of Statistics, University of Minnesota Minneapolis, Minnesota, United States of America; 3 Division of Biostatistics, University of Minnesota School of Public Health, Minneapolis, Minnesota, United States of America; Iran University of Medical Sciences, ISLAMIC REPUBLIC OF IRAN

## Abstract

Network meta-analysis (NMA) expands the scope of a conventional pairwise meta-analysis to simultaneously compare multiple treatments, which has an inherent appeal for clinicians, patients, and policy decision makers. Two recent reports have shown that the impact of excluding a treatment on NMAs can be substantial. However, no one has assessed the impact of excluding a trial from NMAs, which is important because many NMAs selectively include trials in the analysis. This article empirically examines the impact of trial exclusion using both the arm-based (AB) and contrast-based (CB) approaches, by reanalyzing 20 published NMAs involving 725 randomized controlled trials and 449,325 patients. For the population-averaged absolute risk estimates using the AB approach, the average fold changes across all networks ranged from 1.004 (with standard deviation 0.004) to 1.072 (with standard deviation 0.184); while the maximal fold changes ranged from 1.032 to 2.349. In 12 out of 20 NMAs, a 1.20-fold or larger change is observed in at least one of the population-averaged absolute risk estimates. In addition, while excluding a trial can substantially change the estimated relative effects (e.g., log odds ratios), there is no systematic difference in terms of changes between the two approaches. Changes in treatment rankings are observed in 7 networks and changes in inconsistency are observed in 3 networks. We do not observe correlations between changes in treatment effects, treatment rankings and inconsistency. Finally, we recommend rigorous inclusion and exclusion criteria, logical study selection process, and reasonable network geometry to ensure robustness and generalizability of the results of NMAs.

## Introduction

In clinical practice, and at a wider societal level, treatment decisions need to consider all available evidence. Network meta-analysis (NMA) expands the scope of a conventional pairwise meta-analysis to simultaneously compare multiple treatment options [1–4] by collectively synthesizing direct evidence within trials and indirect evidence across trials. In the simplest case, one may be interested in comparing two treatments A and B. Direct evidence comes from randomized controlled trials (RCTs) comparing A and B, while indirect evidence comes from RCTs of either A or B versus a common comparator C. NMA has an inherent appeal for clinicians, patients, and policy decision makers because it enables simultaneous inference of multiple treatments and strengthens inference by including indirect evidence [5].

However, meta-analysts undertaking an NMA often selectively choose trials to include in the systematic reviews due to certain preference. For instance, some NMAs exclude trials with placebo or no treatment due to the belief that the placebo or no treatment may vary over time or be set in favorable conditions to appease regulatory authorities [6]; whereas some NMAs exclude trials without a placebo- or no treatment-arm (i.e., exclude trials comparing solely active treatments) [7]. In addition, some NMAs may include only trials available in a particular location or time period for convenience. It is generally difficult and tedious to include all existing trials that meet the inclusion/exclusion criteria due to some technical issues (i.e., some trials may be published using other languages) in NMA. Intuitively, if the omitted trials are similar to the included trials and there is sufficient number of included trials, the failure to include these omitted trials will only result in less information (i.e., bigger standard errors and wider confidence intervals), but will not have any systematic impact on the estimates. However, if the omitted trials happen to be different from the included, or if the number of included trials is too small to provide robust estimation, then omission of these trials may have profound influence. The exploration of impact of exclusion of trials helps make better sense of a network meta-analysis and guide future design and conduct of trials and meta-analyses.

A recent publication by Mills et al. [8] investigated the impact of removing a treatment arm (including placebo / no treatment) on the estimated effect sizes for NMAs by reanalyzing 18 NMAs, and concluded that excluding a treatment could have substantial influence on estimated effect sizes. They consequently stated that selection of treatment arms should be carefully considered when applying NMAs. Another publication by Lin et al. [9] further explored the sensitivity to excluding treatments using both the *armed-based* (AB) [1] and *contrast-based* (CB) [2] NMA approaches. They found that when a treatment was removed under the CB framework, it was also necessary to exclude the other treatment in two-arm studies that investigated the excluded treatment, while such additional exclusions were not necessary in the AB framework. To the best of our knowledge, no previous works, thus far, have empirically studied the impact of removing a trial in NMAs.

The primary objective of this article is to obtain empirical evidence of the impact of removing a trial on the effect size estimates. We investigate both the AB [1, 4] and CB [3] (a more general version than in [2]) NMA approaches by reanalyzing 20 published NMAs with binary outcomes. The impact on treatment rankings and inconsistency between direct and indirect evidence are also assessed based on the AB approach. This article is organized as follows. First, we describe the characteristics of the 20 network meta-analyses. Second, we briefly introduce the two NMA approaches and our procedures assessing the impact of excluding a trial. Fold changes are used in evaluating the impact on estimated population-averaged absolute risks from the AB approach, and changes in log odds ratios (log *OR*s) are used to compare the results from the AB and CB approaches. We close with a brief discussion with some suggestions for future conduct of NMAs and several limitations of our empirical study.

## Materials and Methods

### Data source and extraction

We reviewed the NMAs studied by Veroniki et al. [10], which searched in PubMed for articles published between March 1997 and February 2011 in which any form of indirect comparison was applied, according to the articles’ titles or abstracts. The authors initially identified 817 articles and after the screening process they ended up with 40 networks. They screened the articles according to 1) whether the networks include at least four treatments, (2) whether the networks contain one closed loop, (3) whether indirect comparisons are included, (4) whether the major outcomes are dichotomous, (5) whether the articles are research papers instead of discussing / commentary papers. We selected 20 networks in our analysis. Nineteen of them were excluded according to our inclusion criterion that each treatment should be compared in at least two trials; otherwise, the networks are poorly connected at that treatment node. Furthermore, a treatment that is only compared in one trial would disappear from the sensitivity analysis if that trial is excluded, disabling the possibility to investigate the impact on any effect sizes related to that specific treatment. A network by Brown et al. [11] was also excluded because zero events were observed in many arms, which would bring bias proportional to the rarity of the event under study [12, 13]. Finally 20 networks involving 725 randomized controlled trials and 449,325 patients were selected; they are Ara 2009 [14], Baker 2009 [15], Ballesteros 2005 [16], Bansback 2009 [17], Bucher 1997 [18], Cipriani 2009 [19], Eisenberg 2008 [20], Elliott 2007 [21], Govan 2009 [22], Lu 2006 [3], Lu 2009 [2], Macfayden 2005 [23], Middleton 2010 [24], Mills 2009 [25], Picard 2000 [7], Puhan 2009 [26], Thijs 2008 [27], Trikalinos 2009 [28], Wang 2010 [29], and Yu 2006 [30].

Table 1 presents the characteristics of the individual networks. Specifically, the first column in Table 1 lists the IDs of these networks. The second column shows the author and year of publication for each NMA. The third to the fifth columns list the type of diseases, the primary outcomes of interest, and the multiple investigated treatments (and their abbreviations) studied in each network. We had preference of efficacy outcome over others for studies that considered more than one outcome, as was done in Veroniki et al. [10]. The sixth column presents the number of trials and treatments contained in each network, from which we can see that each NMA has four or more treatments and more than twice as many studies as treatments. Networks range in size from 9 trials on 4 treatments to 111 trials on 12 treatments. The last column shows the minimum and maximum frequencies for treatments (i.e., the number of trials that contain a treatment) for each network. For example, in the first network Ara 2009 [14], treatments are compared in at least 3 but no more than 7 trials. The frequencies across all networks range from 2 to 89.

**Table 1:** Characteristics of the 20 network meta-analyses.

ID	Network*	Condition/Disease	Outcome	Treatment names (abbreviations)	No. of trials (treatment)	Frequency (min/max)†
1	Ara 2009 [14]	Hypercholesterolaemia	Effectiveness in reducing LDL-c.	1 **Placebo**; 2 Simvastatin 40 mg/day (**SIM 40**); 3 Atorvastatin 80 mg/day (**ATO 80**); 4 Simvastatin 80 mg/day (**SIM 80**); 5 Rosuvastatin 40 mg/day (**ROS 40**)	11 (5)	3/7
2	Baker 2009 [15]	Chronic obstructive pulmonary disease (COPD)	Exacerbation episodes in Chronic Obstructive Pulmonary Disease (COPD> = 1)	1 **Placebo**; 2 Fluticasone (**FLU**); 3 Budesonide (**BUD**); 4 Salmeterol (**SAL**); 5 Formoterol (**FOR**); 6 Tiotropium (**TIO**); 7 Fluticasone+Salmeterol (**FLU+SAL**); 8 Budesonide+Formoterol (**BUD+FOR**)	38 (8)	2/34
3	Ballesteros 2005 [16]	Dysthymia	Efficacy of antidepressants in dysthymia	1 **Placebo**; 2 Tricyclic antidepressant (**TCA**); 3 Selective serotonin reuptake inhibitor (**SSRI**); 4 Monoamine oxidase inhibitor (**MAOI**)	9 (4)	3/9
4	Bansback 2009 [17]	Moderate to severe plaque psoriasis	Efficacy—PASI 75 response score for the treatment of psoriasis	1 **Placebo**; 2 Etanercept (**ETA**); 3 Infliximab (**INF**); 4 Adalimumab (**ADA**); 5 Efalizumab (**EFA**); 6 Alefacept (**ALE**); 7 Cyclosporine (**CYC**); 8 Methotrexate (**MET**)	22 (8)	2/21
5	Bucher 1997 [18]	Pseudocystis carinii	Number of Pseudocystis Carinii pneumonia (prophylaxis against Pneumocystis carinii in HIV infected patients)	1 Aerosolized pentamidine (**AP**); 2 Trimethoprim-sulphamethoxazole (**TMP-SMX**); 3 Dapsone (**D**); 4 Dapsone/pyrimethamine (**D/P**)	18 (4)	4/14
6	Cipriani 2009 [19]	Unipolar major depression	Efficacy—the proportion of patients who responded to the allocated treatment	1 Fluoxetine (**FLU**); 2 Sertraline (**SER**); 3 Citalopram (**CIT**); 4 Eescitalopram (**ESC**); 5 Paroxetine (**PAR**); 6 Fluvoxamine (**FVX**); 7 Milnacipran (**MIL**); 8 Venlafaxine (**VEN**); 9 Reboxetine (**REB**); 10 Bupropion (**BUP**); 11 Mirtazapine (**MIR**); 12 Duloxetine (**DUL**)	111 (12)	6/52
7	Eisenberg 2008 [20]	Smoking	Smoking abstinence	1 **Placebo**; 2 Buprobion (**BUP**); 3 Nicotine gum (**NG**); 4 Transdermal nicotine (**TN**); 5 Varenicline (**VAR**)	61 (5)	6/61
8	Elliott 2007 [21]	Hypertension	Effect of antihypertensives on incidence diabetes mellitus-proportion of patients who developed diabetes	1 **Placebo**; 2 Thiazide diuretic (**TD**); 3 Angiotensin- converting enzyme (**ACE**) inhibitor; 4 Calcium-channel blockers (**CCB**); 5 Angiotensinreceptor blockers (**ARB**); 6 *β*-blocker (**BB**)	22 (6)	5/9
9	Govan 2009 [22]	Stroke	Death by the end of scheduled follow up	1 Stroke ward (**SW**); 2 General medical ward (**GMW**); 3 Mixed rehabilitation ward (**MRW**); 4 Mobile stroke team (**MST**); 5 Acute (semi-intensive) ward (**AW**)	28 (5)	2/24
10	Lu 2006 [3]	Smoking	Smoking cessation	1 **No contact**; 2 **Self-help**; 3 Inidividual counselling (**IC**); 4 Group counselling (**GC**)	24 (4)	6/19
11	Lu 2009 [2]	Gastroesophageal reflux disease	The number of healed patients at one or more follow-up times	1 **Placebo**; 2 Prokinetic agents (**PA**); 3 H_2_ receptor antagonists (**H2RA**); 4 H2RA double dose (**H2RA-D**); 5 Proton pump inhibitors (**PPI**); 6 PPI double dose (**PPI-D**)	40 (6)	4/32
12	Macfayden 2005 [23]	Chronically discharging ears with underlying eardrum perforations	Resolution of discharge	1 No treatment (**No Trt**); 2 Topical quinolone antibiotic (**TQA**); 3 Topical non-quinolone antibiotic (**TNQA**); 4 Topical antiseptic (**TA**)	13 (4)	2/11
13	Middleton 2010 [24]	Heavy menstrual bleeding	Efficacy as second line treatment for heavy menstrual bleeding—dissatisfaction at 12 months	1 “First generation” endometrial destruction techniques (**FG**); 2 Hysterectomy (**HYST**); 3 “Second generation” endometrial destruction techniques (**SG**); 4 Mirena (**MIR**)	20 (4)	4/17
14	Mills 2009 [25]	Smoking	Smoking Abstinence at approximately 4 weeks post-target quit date (TQD)	1 **Control**; 2 Nicotine replacement therapy (**NRT**); 3 Bupropion (**BUP**); 4 Varenicline (**VAR**)	89 (4)	9/89
15	Picard 2000 [7]	Pain on injection	Analgecic efficacy of proplylactic interventions for the prevention of pain on injection with propofol—no pain	1 **Placebo**; 2 No treatment (**No Trt**); 3 Lidocaine (mg) given before the injection of propofol (**LIDb**); 4 Lidocaine (mg) mixed with propofol 200 mg (**LIDm**); 5 Lidocaine (mg) with tourniquet (**LID+TOU**); 6 Opioids (**OPI**); 7 Metoclopramide (**MET**); 8 Temperature (**TEM**).	43 (8)	4/34
16	Puhan 2009 [26]	Chronic obstructive pulmonary disease (COPD)	Exacerbation in patients with chronic obstructive pulmonary disease	1 **Placebo**; 2 Long-acting beta-agonists (**BA**); 3 Long-acting anticholinergics (**AC**); 4 Inhaled corticosteroids (**IC**); 5 Combined treatment with a long-acting beta-agonist and an inhaled corticosteroid (**CT**)	34 (5)	8/31
17	Thijs 2008 [27]	Stroke	Efficacy of antiplatelet in the prevention of serious vascular events after transient ischaemic attack or stroke	1 **Placebo**; 2 Thienopyridines (ticlopidin or clopidogrel) + Aspirin (**THI+ASA**); 3 **ASA**; 4 Aspirin and dipyridamole (**ASA+DP**); 5 **THI**	23 (5)	3/19
18	Trikalinos 2009 [28]	Coronary artery disease	Coronary artery disease—death	1 Medical therapy (**MT**); 2 Percutaneous transluminal balloon coronary angioplasty (**PTCA**); 3 Bare-metal stents (**BMS**); 4 Drugeluting stents (**DES**).	62 (4)	13/52
19	Wang 2010 [29]	Catheter-related infections	Catheter colonisation	1 **Standard**; 2 Chlorhexidine and silver sulfadiazine + (**CHSS+**); 3 Benzalkonium chloride (**BC**); 4 Silver iontophoretic (**SIT**); 5 Minocycline-rifampicin (**MI**); 6 **CHSS**; 7 Silver alloy-coated (**SAC**); 8 Silver-impregnated (**SIP**); 9 Heparin-bonded (**HB**)	43 (9)	2/40
20	Yu 2006 [30]	Cardiac surgery	Cardiac ischemic complications and mortality	1 **Control**; 2 Enflurane (**ENF**); 3 Isoflurane (**ISO**); 4 Halothane (**HAL**); 5 Sevoflurane (**SEV**); 6 Desflurane (**DES**)	14 (6)	2/14

* Network shows the author and year of each published NMA.

^†^ Frequency reports the smallest and largest number of trials that contain a treatment in each network.

**Fig 1:**
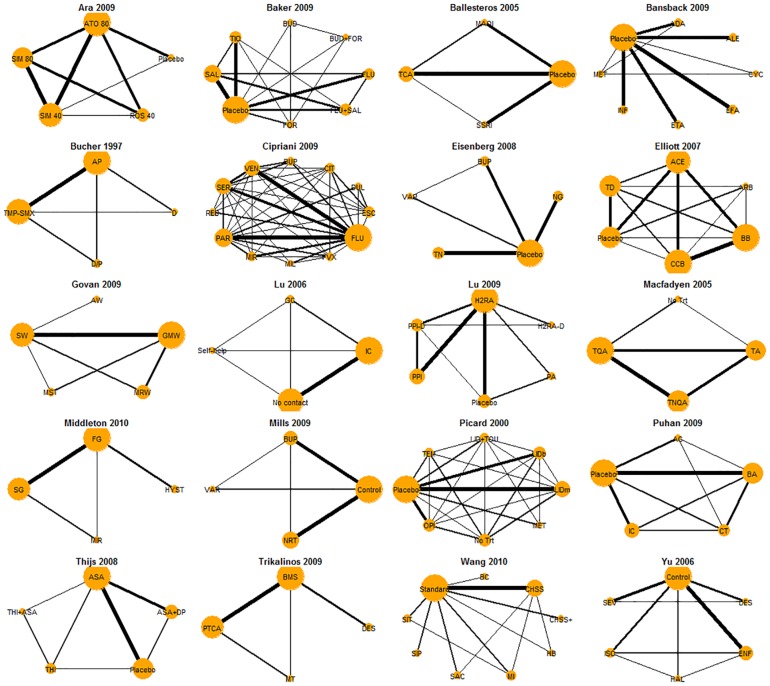
Graphacial representations for the 20 NMAs. The thickness of each link is proportional to the number of trials investigaing the relation, and the size of each treatment node is proprotional to the number of direct comparisons that contain that treatment.

Fig 1 graphically displays the 20 networks. In each network plot, the thickness of each link is proportional to the number of trials investigaing the relation, and the size of each treatment node is proprotional to the number of direct comparisons that contain that treatment. Neither the number of trials for each pairwise comparison nor the number of direct comparisons for each treatment are balanced in all networks. The pool includes various constructions of networks, where one of which (i.e., Lu 2006 [3]) contains direct information for all pairwise comparisions while the rest do not.

### Statistical models for NMA with binary data

Now, we briefly introduce both the AB and CB approaches using Bayesian hierarchical models. The AB approach focuses on absolute risks for each treatment arm, while the CB approach focuses on relative effects (e.g., ORs under binary case). Existing literature [1–4, 31, 32] has explored and discussed the model assumptions and model fit of the two approaches, and two recent discussion papers have further provided detailed comparisons on their strengths and limitations; see [33, 34].

We consider an NMA of *I* trials and *K* treatments of interest. Since most of the trials only compare a subset of the treatments of interest, we let *S*_*i*_ denote the set of treatments that are compared in the *i*^*th*^ trial, whose cardinality is equal to or smaller than (in most cases) *K*. Let *n*_*ik*_ be the total number of subjects, *y*_*ik*_ be the number of events, and *p*_*ik*_ be the corresponding probability of events for the *k*^*th*^ treatment in the *i*^*th*^ trial. We denote all observed data by *D*.

For the AB approach proposed by Zhang et al. [1, 4], it specifies *y*_*ik*_~*Bin*(*n*_*ik*_, *p*_*ik*_), *k*∈*S*_*i*_, *i* = 1,…,*I*, and Φ^−1^(p_*ik*_) = *μ*_*k*_ + *σv*_*ik*_, (*v*_*i*1_,…,*v*_*ik*_)^*T*^ ~ *MVN*(0, R_*K*_). Here *μ*_*k*_ is the fixed treatment effect for the *k*^*th*^ treatment, *σ* is the standard deviation for the random effects *v*_*ik*_, and R_*K*_ is an exchangeable correlation matrix. The population-averaged treatment-specific event rate *π*_*k*_ has a closed form based on the above model: $\pi_{k}=E\left(p_{ik}|\mu_{k},\sigma \right)=\int_{-\infty }^{+\infty }\Phi \left(\mu_{k}+\sigma z\right)\phi \left(z\right)dz=\Phi \left(\mu_{k}/\sqrt{1+\sigma^{2}}\right),\;k=1,\ldots ,\;K$, where *ϕ*() is the density function and Φ() is the cumulative density function of the standard normal distribution. The ranking of treatments is calculated based on *π*_*k*_. When the outcome has a positive interpretation (say, efficacy), the posterior *probability of being the best* (Pbest) is *P*(*k* is the best treatment | *D*) = *P*(rank(*π*_*k*_) = 1 | *D*); while when the outcome has a negative interpretation (say, adverse event), it is *P*(*k* is the best treatment | *D*) = *P*(rank(*π*_*k*_) = *K* | *D*). The marginal ORs are then defined as *OR*_*kl*_ = [*π*_*k*_/(1 − *π*_*k*_)]/[*π*_*l*_/(1 − *π*_*l*_)] for a pairwise comparison between Treatments *k* and *l* (*k*≠*l*). We report ORs in addition to event rates in this paper in order to be consistent with the CB approach.

Zhao et al. [35] proposed methods to detect inconsistency based on the AB approach. To measure inconsistency between Treatments *k*_1_ and *k*_2_, trials are divided into four groups: (i) trials that include both *k*_1_ and *k*_2_, (ii) trials include *k*_1_ but not *k*_2_, (iii) trials include *k*_2_ but not *k*_1_, (iv) trials that include neither *k*_1_ nor *k*_2_. Then the discrepancy of direct and indirect evidence can be tested by computing the posterior distribution of the discrepancy factor ${△}_{k_{1}k_{2}}=(\mu_{k_{1}}^{\left(i\right)}-\mu_{k_{2}}^{\left(i\right)})-(\mu_{k_{1}}^{\left(ii\right)}-\mu_{k_{2}}^{\left(iii\right)})$. If zero is in the far tail of this posterior distribution, then inconsistency is found. Note that each pair of treatments needs to be assessed in a separate model and a pair with no information of group (i), (ii) or (iii) is ineligible for inconsistency detection.

For the CB approach proposed by Lu & Ades [3], it is based on the following hierarchical specification: logit(*p*_*ik*_) = *μ*_*i*_ + *X*_*ik*_*δ*_*ib*(i)k_, where *μ*_*i*_ is the baseline effect in Trial *i*, *X*_*ik*_’s are indicators for baseline treatments taking value 0 if k = b and 1 if k≠b, and *δ*_*ib*(i)k_ represents the relative random effect of Treatment k versus b(i) on log odds scale in the *i*^*th*^ trial. In the next step, the vector (*δ*_*ib*(i)k_) is assumed to have a |*S*_*i*_| − 1 dimensional normal distribution (a univariate normal distribution if the *i*^*th*^ trial contains two arms or a multivariate normal distribution if the *i*^*th*^ trial contains multiple arms) with mean vector (*d*_*b*(i)k_) and covariance matrix $V_{\left|S_{i}-1\right|}$, i.e., $\left(\delta_{ib(i)\text{k},\text{k}\in S_{i}})\sim MVN(d,V_{\left|S_{i}-1\right|}\right)$. A very common $V_{\left|S_{i}-1\right|}$ is a homogeneous-variance exchangeable matrix with correlation 1/2, i.e., *δ*_*ib*(i)k_ ~ *N*(*d*_*b*(i)k_, *σ*^2^) and cov(*δ*_*ib*(i)k_, *δ*_*ib*(i)h_) = *σ*^2^/2. The model in addition assumes exchangeability, i.e., *d*_*kl*_ = *d*_*bk*_ − *d*_*bl*_. Finally $OR_{kl}=e^{d_{kl}}$.

### Sensitivity analysis of excluding a trial

Regardless of the approaches used in the original publications of the 20 NMAs, we reanalyzed them in this paper using both the AB and CB approaches described in the previous Section. Five steps were applied to each NMA to analyze the impact of omission of trials on the estimated treatment effects and two more steps were conducted to assess the influence on treatment ranks and inconsistency. The details are as follows:

1. Fit the AB and CB NMA Bayesian hierarchical models separately to the complete data of each NMA. For the AB approach, both absolute risks for each treatment arm and ORs for all pairwise comparisons are recorded; while for the CB approach, only ORs are recorded.
2. Remove each trial within each NMA and reanalyze the data using both the AB and CB approaches. Same statistical summaries are recorded as in Step 1.
3. Calculate *fold changes* in the estimated absolute risks (from the AB approach) to evaluate the impact of exclusion of trials. The fold change for Treatment *k* from omission of Trial *i* is equal to $\pi_{k}^{\left(i\right)}/\pi_{k}$ if $\pi_{k}^{\left(i\right)}/\pi_{k}>1$; otherwise it is equal to $\pi_{k}/\pi_{k}^{\left(i\right)}$. Here $\pi_{k}^{\left(i\right)}$ is the estimated absolute risk for Treatment *k* after exclusion of Trial *i*. In other words, fold changes are always expressed as a value greater than 1.00. Take a simple example, if a specific event rate is 0.70 in the full network and 0.50 in the network with one trial excluded, then the change is 0.70/0.50 = 1.40-fold; if the event rates are 0.40 and 0.60 instead in the full and incomplete networks respectively, then the change is 0.60/0.40 = 1.50-fold. The larger the fold change is, the larger the impact is.
4. Compute the log *OR* changes after excluding a trial using the AB and CB approaches (i.e., $\text{log}\frac{OR_{AB}^{\left(\text{i}\right)}}{OR_{AB}}$ and $\text{log}\frac{OR_{CB}^{\left(\text{i}\right)}}{OR_{CB}}$, respectively). Here $OR_{AB}^{\left(\text{i}\right)}$ and $OR_{CB}^{\left(\text{i}\right)}$ represent the ORs estimated from the AB and CB approaches without Trial *i*. If $\text{log}\frac{OR_{AB}^{\left(\text{i}\right)}}{OR_{AB}}$ and $\text{log}\frac{OR_{CB}^{\left(\text{i}\right)}}{OR_{CB}}$ are around 0, then there is subtle impact of excluding Trial *i*. The further they are from 0, the larger the impact of excluding Trial *i* is.
5. Compare the difference between $\text{log}\frac{OR_{AB}^{\left(\text{i}\right)}}{OR_{AB}}$ and $\text{log}\frac{OR_{CB}^{\left(\text{i}\right)}}{OR_{CB}}$ through graphical tools (e.g., scatter plot and Bland-Altman plot) and statistical tests. The average of $\left|\text{log}\frac{OR_{AB}^{\left(\text{i}\right)}}{OR_{AB}}\right|$ of all pairwise comparisons using all eligible trial exclusions across all networks from the AB approach is compared with that from the CB approach (i.e., the average of $\left|\text{log}\frac{OR_{CB}^{\left(\text{i}\right)}}{OR_{CB}}\right|$). Bootstrap resampling technique [36] is applied to compute the 95% confidence intervals (CIs) and the *p*-value for testing difference. Note that 10,000 bootstrap samples are constructed at the network level; that is, each sample contains 20 resampled networks, drawn with replacement from the original 20 networks.
6. Assess whether the best treatment and the corresponding Pbest of that treatment change after omission of trials, using the AB approach.
7. Evaluate the influence of omission of trials on inconsistency using the AB approach.

Step 3 evaluates the impact of omission of trials on the estimated absolute risks using the AB approach, and Steps 4 and 5 compare the results of impact based on the AB and CB approaches. Steps 1–5 investigate the impact on treatment effects, while Steps 6–7 further explore the influence on treatment ranks and inconsistency.

Analyses were conducted via Markov chain Monte Carlo (MCMC) methods using JAGS [37] and the R package “rjags” [38]. The S1 Appendix provides the JAGS codes for both approaches. The convergence of the MCMC chains was assessed by the Gelman-Rubin convergence statistic [39] and a visual inspection of the chains.

## Results

### Fold changes in event rates estimated from the AB approach

The average and maximal fold changes for each network from the AB approach are reported in Table 2. The average fold changes across all networks ranged from 1.004 (with standard deviation 0.004) to 1.072 (with standard deviation 0.184); while the maximal fold changes ranged from 1.032 to 2.349. In 8 of 20 networks, the maximal changes were below 1.200-fold; while 5 of them obtained maximal changes below 1.100-fold. Mills et al. [8] suggested considering relative changes exceeding 1.20-fold as substantial. Using this threshold, 12 out of the 20 networks had relative changes larger than 1.20-fold observed in at least one of the population-averaged absolute risk estimates. It suggests that omission of trials may have substantial impact on the estimation.

**Table 2:** Summary of fold changes in terms of estimated event rates using the AB approach.

Network	Fold Change		Proportions of fold changes within each magnitude category*					
	Average (sd)	Maximal	1.00–1.10	1.10–1.20	1.20–1.30	1.30–1.40	1.40–1.50	>1.50
Ara 2009 [14]	1.054 (0.049)	1.215	0.836	0.145	*0*.*018*	0.000	0.000	0.000
Baker 2009 [15]	1.025 (0.030)	1.388	0.987	0.010	0.000	*0*.*003*	0.000	0.000
Ballesteros 2005 [16]	1.038 (0.034)	1.174	0.972	0.028	0.000	0.000	0.000	0.000
Bansback 2009 [17]	1.033 (0.099)	1.835	0.949	0.028	*0*.*006*	0.000	0.000	*0*.*017*
Bucher 1997 [18]	1.044 (0.052)	1.219	0.861	0.125	*0*.*014*	0.000	0.000	0.000
Cipriani 2009 [19]	1.004 (0.004)	1.057	**1.000**	0.000	0.000	0.000	0.000	0.000
Eisenberg 2008 [20]	1.010 (0.009)	1.057	**1.000**	0.000	0.000	0.000	0.000	0.000
Elliott 2007 [21]	1.034 (0.033)	1.130	0.909	0.091	0.000	0.000	0.000	0.000
Govan 2009 [22]	1.028 (0.117)	2.349	0.986	0.000	0.000	*0*.*007*	0.000	*0*.*007*
Lu 2006 [3]	1.028 (0.040)	1.241	0.948	0.042	*0*.*010*	0.000	0.000	0.000
Lu 2009 [2]	1.016 (0.035)	1.329	0.962	0.033	0.000	*0*.*004*	0.000	0.000
Macfayden 2005 [23]	1.034 (0.038)	1.156	0.904	0.096	0.000	0.000	0.000	0.000
Middleton 2010 [24]	1.035 (0.048)	1.338	0.938	0.050	0.000	*0*.*012*	0.000	0.000
Mills 2009 [25]	1.006 (0.005)	1.032	**1.000**	0.000	0.000	0.000	0.000	0.000
Picard 2000 [7]	1.013 (0.020)	1.222	0.988	0.009	*0*.*003*	0.000	0.000	0.000
Puhan 2009 [26]	1.028 (0.019)	1.093	**1.000**	0.000	0.000	0.000	0.000	0.000
Thijs 2008 [27]	1.018 (0.013)	1.044	**1.000**	0.000	0.000	0.000	0.000	0.000
Trikalinos 2009 [28]	1.020 (0.030)	1.336	0.988	0.008	0.000	*0*.*004*	0.000	0.000
Wang 2010 [29]	1.017 (0.035)	1.498	0.982	0.013	*0*.*003*	0.000	*0*.*003*	0.000
Yu 2006 [30]	1.072 (0.184)	2.303	0.881	0.036	*0*.*012*	*0*.*048*	0.000	*0*.*024*

* sd represents standard deviation. Cells are in bold if all fold changes in the network fall in [1.00, 1.10]; cells are italic if fold changes > 1.20. 1.00–1.10 = [1.00, 1.10]; 1.10–1.20 = (1.10, 1.20]; 1.20–1.30 = (1.20, 1.30]; 1.30–1.40 = (1.30, 1.40]; 1.40–1.50 = (1.40, 1.50].

Table 2 also summarizes the proportions of fold changes in the estimated event rates falling in [1.00, 1.10], (1.10, 1.20], (1.20, 1.30], (1.30, 1.40], (1.40, 1.50], and (1.50, +∞) intervals for the 20 NMAs. Five networks, which were Cipriani 2009 [19], Eisenberg 2008 [20], Mills 2009 [25], Puhan 2009 [26], and Thijs 2008 [27], obtained fold changes of estimated event rates all smaller than 1.10-fold. Fold changes in another three networks, Ballesteros 2005 [16], Elliott 2007 [21], and Macfayden 2005 [23], were all smaller than 1.20 with some larger than 1.10. Nine networks obtained relative changes all smaller than 1.50-fold with some exceeding 1.20-fold; they were Ara 2009 [14], Baker 2009 [15], Bucher 1997 [18], Lu 2006 [3], Lu 2009 [2], Middleton 2010 [24], Picard 2000 [7], Trikalinos 2009 [28] and Wang 2010 [29]. The rest three networks, i.e., Bansback 2009 [17], Govan 2009 [22] and Yu 2006 [30], contained changes in estimated event rates larger than 1.50-fold.

We further explore the features of the three networks with fold changes larger than 1.50. In Bansback 2009 [17], exclusion of Trials 21 and 22 led to 1.805-fold and 1.835-fold changes in the estimated event rate for Treatment 7 (Cyclosporine), respectively. This observation is understandable because Trials 21 and 22 were the only two trials containing Cyclosporine, whereas the crude event rates (observed number of events / observed total number of subjects) of Cyclosporine in the two trials were 0.200 and 0.714, respectively. Thus excluding either trial would lead to substantial changes in estimation. In addition, exclusion of Trial 10 in this network resulted in a 1.541-fold change in event rate for Treatment 8 (Methotrexate), which were compared in only Trials 10 and 22 with sample sizes 110 and 43 and crude event rates 0.364 and 0.605, respectively. In Govan 2009 [22], Treatment 5 (Acute ward) was compared in only Trials 25 and 26, and the exclusion of Trial 26 resulted in a fold change of value 2.349 in the estimated event rate for Acute ward. Though crude event rates in those two trials were not significantly different, Trial 26 contained a much larger sample size of 134 in contrast to 27 for Trial 25. In Yu 2006 [30], Treatment 4 (Halothane) was compared in only Trials 1 and 8 with sample sizes 253 and 14 and crude event rates 0.036 and 0.071, and Treatment 6 (Desflurane) was compared in only Trials 11, 13 and 14 with sample sizes 80, 100, 25 and crude event rates 0.013, 0.040 and 0.000. The exclusion of Trials 1 and 13 produced 1.951-fold and 2.303-fold changes in the estimated event rates for Treatments 4 and 6, respectively. In summary, the most influential trials typically contain larger sample sizes among the few trials that compare treatments with small frequencies (in other words, treatments that are compared in small numbers of trials) and sometimes report different crude event rates from the rest. Omission of those trials may bring larger impact in the estimation of treatment effects, thus may influence treatment comparison and decision making. It further implies the importance of network geometry.

### Comparison of the results from the AB and CB approaches

**Fig 2:**
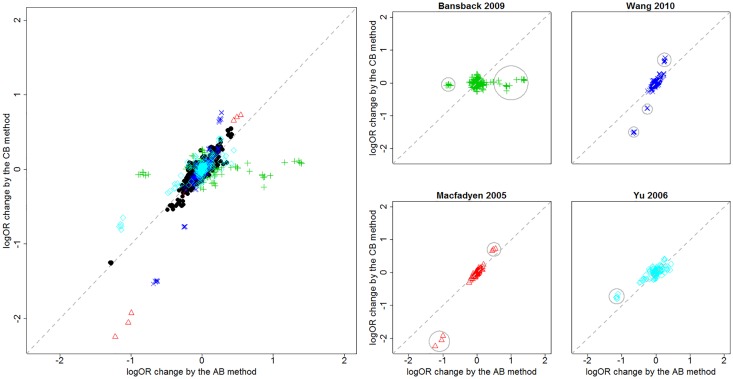
Scatter plots of logOR changes after and before excluding trials for the 20 networks. The x-axis labels changes obtained from the AB approach, i.e., $\text{log}\frac{OR_{AB}^{\left(\text{i}\right)}}{OR_{AB}}$, while the y-axis labels changes obtained from the CB approach, i.e., $\text{log}\frac{OR_{CB}^{\left(\text{i}\right)}}{OR_{CB}}$. Left panel pools results from the 20 networks with scatters deviating from the identity line in color. Right panel excerpts colored scatters.

$\text{log}\frac{OR_{CB}^{\left(\text{i}\right)}}{OR_{CB}}$ and $\text{log}\frac{OR_{AB}^{\left(\text{i}\right)}}{OR_{AB}}$ were recorded and used to compare the performance of the AB and CB approaches. The left panel in Fig 2 presents the scatter plots of $\text{log}\frac{OR_{CB}^{\left(\text{i}\right)}}{OR_{CB}}$ against $\text{log}\frac{OR_{AB}^{\left(\text{i}\right)}}{OR_{AB}}$ pooled from the 20 networks across all pairwise comparisons and trial exclusions. Most of the scatters tended to concentrate in the vicinity of the identity line, i.e., a *y* = *x* line, suggesting agreement between the AB and CB approaches. But scatters from four networks, i.e. Bansback 2009 [17], Macfadyen 2005 [23], Wang 2010 [29] and Yu 2006 [30], were found to deviate from the identity line and marked with colored points. The right panel excerpts scatter plots for these four networks individually. For Bansback 2009 [17] and Yu 2006 [30], omission of trials had larger impact from the AB approach than from the CB approach; while for Macfadyen 2005 [23] and Wang 2010 [29], CB approach was more sensitive to excluding trials. However, only small numbers of the points in the scatter plots were away from the identity line; more specifically, 22 out of 616 (i.e., 3.6%) in Bansback 2009 [17], 6 out of 78 (i.e., 7.7%) in Macfadyen 2005 [23], 5 out of 210 (i.e., 2.4%) in Wang 2010 [29] and 24 out of 1548 (i.e., 1.6%) in Yu 2006 [30]. These points are circled in their individual scatter plots in the right panel of Fig 2.

**Fig 3:**
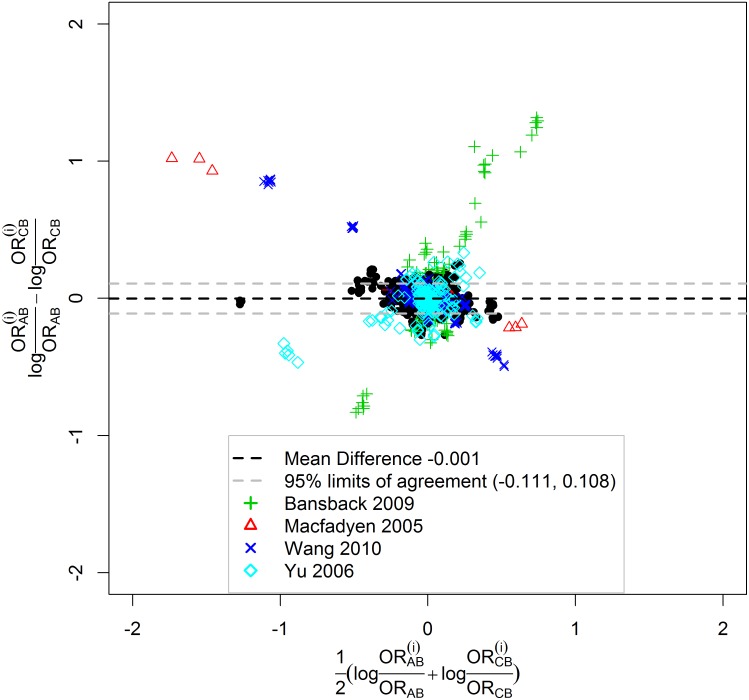
Bland-Altman plot. The differences between the AB and CB approaches in terms of log OR changes after omission of trials, i.e., $\text{log}\frac{OR_{AB}^{\left(\text{i}\right)}}{OR_{AB}}-\text{log}\frac{OR_{CB}^{\left(\text{i}\right)}}{OR_{CB}}$, is drawn agaist the mean, i.e., $\frac{1}{2}(\text{log}\frac{OR_{AB}^{\left(\text{i}\right)}}{OR_{AB}}+\text{log}\frac{OR_{CB}^{\left(\text{i}\right)}}{OR_{CB}})$. The mean difference and 95% limits of agreement are shown in dashed lines. Four networks are highlighted in color.

The Bland-Altman plot in Fig 3 further consolidates the agreement between these two approaches on the impact of excluding trials. The differences $\text{log}\frac{OR_{AB}^{\left(i\right)}}{OR_{AB}}-\text{log}\frac{OR_{CB}^{\left(i\right)}}{OR_{CB}}$ pooled from all networks including all pairwise comparisons and trial exclusions were plotted against the means $\frac{1}{2}(\text{log}\frac{OR_{AB}^{\left(i\right)}}{OR_{AB}}+\text{log}\frac{OR_{CB}^{\left(i\right)}}{OR_{CB}})$. The mean of these differences (i.e., mean of $\text{log}\frac{OR_{AB}^{\left(i\right)}}{OR_{AB}}-\text{log}\frac{OR_{CB}^{\left(i\right)}}{OR_{CB}}$) was equal to -0.001 and was drawn in black dashed line in Fig 3. The standard deviation (SD) of the differences was 0.055 and the width of the 95% limits of agreement (drawn in grey dashed lines) was 0.219. The narrow range of the 95% limits of agreement showed good agreement. In addition, 98.2% (15597/15878) of the differences were contained in the 95% limits of agreement. Thus we conclude that the CB approach agrees well with the AB approach in terms of the impact of excluding trials. Note that the 4 excerpted networks are also highlighted in color in the Bland-Altman plot.

Statistical testing was also conducted to compare the AB and CB approaches in addition to the graphical exploration. We let *η*_*AB*_ and *η*_*BC*_ denote the true mean $\left|\text{log}\frac{OR_{AB}^{\left(\text{i}\right)}}{OR_{AB}}\right|$ and mean $\left|\text{log}\frac{OR_{CB}^{\left(\text{i}\right)}}{OR_{CB}}\right|$ of all pairwise comparisons and trial exclusions from all networks for the AB and CB approaches, respectively. The estimates for *η*_*AB*_ and *η*_*BC*_ based on the current data were 0.021 and 0.021. Using the 10,000 bootstrap samples, 95% CIs for *η*_*AB*_ and *η*_*BC*_ were estimated to be (0.014, 0.040) and (0.013, 0.038), respectively. For the hypothesis testing *H*_0_:*η*_*AB*_ = *η*_*BC*_ versus *H*_*A*_: *η*_*AB*_ ≠ *η*_*BC*_, the *p*-value was calculated based on another 10,000 bootstrap samples under the null hypothesis. It turned out that *p*-value = 0.156. Therefore the absolute log *OR* changes under the AB approach were not statistically significantly different from those under the CB approach.

### Impact on treatment ranks and inconsistency based on the AB approach

Table 3 shows changes in the best treatment and Pbest after omission of trials. Networks whose outcomes have negative interpretations are listed in italics. The best treatment in thirteen networks after omission of trials remains the same. The Pbest of that treatment is also provided for both the full and reduced networks. For example, in Ara 2009 [14], ATO 80 ranks as the best treatment in both the full (with Pbest = 0.880) and reduced networks (with Pbest ranging from 0.778 to 0.878). The rest seven networks show changes in the best treatment. For Baker 2009 [15], BUD + FOR is the best treatment in the full network with Pbest = 0.463, while TIO is the best treatment after omission of Trials 11, 16, 17, 22, 26 or 34 with Pbest = 0.470, 0.551, 0.448, 0.479, 0.514 and 0.445 respectively, and BUD is the best treatment after omission of Trial 18. For Ballesteros 2005 [16], MAOI is the best treatment with Pbest = 0.496 in the full network, but SSRI becomes the best treatment with Pbest = 0.529 after omission of Trial 18. For Lu 2009 [2], PPI-D is the best treatment in the full network with Pbest = 0.567, but PPI becomes the best after omission of Trials 19, 22, 36 or 39 with Pbest = 0.538, 0.523, 0.584 and 0.538 respectively. For Puhan 2009 [26], AC is the best treatment in the full network with Pbest = 0.545 and CT is the best after omission of Trials 9, 19 or 33 with Pbest = 0.343, 0.405, 0.344 respectively. For Wang 2010 [29], MI is the best treatment with Pbest = 0.619 in the full network but CHSS+ becomes the best with Pbest = 0.518 after omission of Trial 37. Finally for Yu 2006 [30], SEV is the best treatment with Pbest = 0.673 in the full network while DES becomes the best with Pbest = 0.723 after omission of Trial 13.

**Table 3:** Impact on treatment ranks, probability of the best treatment, and inconsistency using the AB approach. Note: For networks in italics, the treatment with the lowest event rate is the best treatment; for the other networks, the treatment with the highest event rate is the best treatment. -----represents that inconsistency cannot be assessed.

Network	Change in the best treatment	Change in probability of being the best treatment		Change in inconsistency
		Full	Reduced	
Ara 2009 [14]	None	ATO 80 (0.880)	ATO 80 (0.778–0.878)	None
*Baker 2009* [15]	Top three switch	BUD+FOR (0.463)	BUD + FOR (0.437–0.546); TIO (0.470, 0.551, 0.448, 0.479, 0.514, 0.445 after omission of Trials 11, 16, 17, 22, 26, 34); BUD (0.796 after omission of Trial 18)	None
Ballesteros 2005 [16]	Top two switch	MAOI (0.496)	MAOI (0.476–0.674); SSRI (0.529 after omission of Trial 6)	-----
Bansback 2009 [17]	None	INF (0.971)	INF (0.815–0.979)	-----
*Bucher 1997* [18]	None	TMP-SMX (0.996)	TMP-SMX (0.961–0.998)	None
Cipriani 2009 [19]	None	MIR (0.541)	MIR (0.381–0.639)	None
Eisenberg 2008 [20]	None	VAR (0.974)	VAR (0.942–0.987)	Yes (inconsistency between BUP and VAR observed after omission of Trial 61)
Elliott 2007 [21]	None	TD (0.698)	TD (0.535–0.858)	None
*Govan 2009* [22]	None	AW (0.987)	AW (0.881–0.993)	None
Lu 2006 [3]	None	GC (0.760)	GC (0.554–0.907)	None
Lu 2009 [2]	Top two switch	PPI-D (0.567)	PPI-D (0.500–0.829); PPI (0.538, 0.523, 0.584, 0.538 after omission of Trials 19, 22, 36, 39)	None
*Macfayden 2005* [23]	None	TQA (0.946)	TQA (0.855–0.967)	None
Middleton 2010 [24]	Top two switch	MIR (0.458)	MIR (0.438–0.849); FG (0.465, 0.556, 0.414, 0.492 after omission of Trials 16, 17, 18, 20)	None
Mills 2009 [25]	None	VAR (0.994)	VAR (0.978–0.997)	None
Picard 2000 [7]	None	LID+TOU (0.870)	LID + TOU (0.713–0.939)	None
*Puhan 2009* [26]	Top two switch	AC (0.545)	AC (0.469–0.675); CT (0.343, 0.405, 0.344 after omission of Trials 9, 19, 33)	None
*Thijs 2008* [27]	None	ASA+DP (0.715)	ASA+DP (0.582–0.802)	Yes (inconsistency between Placebo and ASA observed after omission of Trial 4)
*Trikalinos 2009* [28]	None	DES (0.700)	DES (0.581–0.820)	Yes (Inconsistency between PTCA and BMS disappear after omission of Trials 7, 10, 17, 46, 50, 51, 53, 57, 62)
*Wang 2010* [29]	Top two switch	MI (0.619)	MI (0.430–0.852); CHSS+ (0.518 after omission of Trial 37)	None
*Yu 2006* [30]	Top two switch	SEV (0.673)	SEV (0.487–0.789); DES (0.723 after omission of Trial 13)	-----

Changes in inconsistency are also presented in Table 3. Three networks are not assessed because omission of some trials in these networks loses information of group (i), (ii) or (iii) for all pairs of treatments and thus disables the detection of inconsistency. For the rest seventeen networks, one eligible pair for each network is assessed. Omission of trials does not change the status of inconsistency in most networks except three (Eisenberg 2008 [20], Thijs 2009 [27] and Trikalinos 2009 [28]). In Eisenberg 2008 [20], inconsistency between BUP and VAR is observed after omission of Trial 61. In Thijs 2009 [27], inconsistency between Placebo and ASA appears after omission of Trial 4. In Trikalinos 2009 [28], inconsistency between PTCA and BMS disappears after omission of Trials 7, 10, 17, 46, 50, 51, 53, 57 or 62.

## Discussion

It is common for NMAs to exclude specific trials and treatment arms based on diverse criteria [8], some limitations and preferences. The impact of exclusion of treatments arms was investigated in Mills et al. [8] and Lin et al. [9] empirically and substantial influence was found, whereas the impact of exclusion of trials has not been explored before. In this paper we empirically studied this impact using 20 published networks and documented that exclusion of trials can sometimes affect the estimation of treatment effects substantially.

We also found that exclusion of trials, which contain larger sample sizes compared with the other trials in the comparison of treatments with sparse information and which report different crude event rates from the rest, tend to result in larger changes in the estimation, which is as expected. Broadly network geometry including the abundance of trials, randomized patients for different trials and gaps of evidence in the treatment network should be taken seriously. In addition, the changes in treatment ranks and inconsistency are not correlated with changes in treatment effects.

Although the AB approach focuses on reporting population-averaged absolute risks and the CB approach focuses on estimating ORs, they both are sensitive to excluding trials. Our empirical study suggested that the two approaches generally agreed on the magnitude of changes in log *OR* (i.e.,$\text{log}\frac{OR^{\left(\text{i}\right)}}{OR}$), though some small disagreement were observed in 4 of the 20 networks. This work also contributes to the call for more empirical comparison of the AB and CB approaches [33, 34].

It has been discussed in the literature on how eligibility criteria may influence the results and the conclusions of traditional pairwise meta-analysis [40–44]. These findings suggest that in meta-analysis comparing multiple treatments, it is also very important to develop a rigorous systematic review protocol with logically considered inclusion and exclusion criteria and study selection process, such that the results from NMAs are robust and generalizable.

There are some limitations in our analysis. First, we used a selection criterion requiring each treatment to be studied in at least two studies. The literature has no well-established criterion serving this purpose. Second, though we did check the changes in evidence consistency, inconsistency detection in NMA is still an open question, has problems under both AB and CB framework, and awaits improvements [3, 35]. Third, we did not check outlying trials in this empirical study. Methods may need to be tailored to downweight outlyingness if needed [31].

Turning to future work, we are interested in exploring better inclusion and exclusion criteria for NMAs such as the minimum number of trials required to include a treatment arm in the NMA, and how to account for study quality in NMAs. Sufficient number of trials for each treatment arm is required to ensure sufficient statistical power to make robust conclusion, whereas outlying or low-quality trials should be deleted or down-weighted at the same time [31]. These have the potential to serve as supplement to the guidance for future conduct of NMAs and contribute to the Preferred Reporting Items for Systematic Reviews and Meta-analyses (PRISMA) Extension Statement [45].

## Supporting Information

S1 Appendix JAGS codes for the AB and CB approaches. (PDF)
