# Supplementary material for: The Impact of Excluding Trials from Network Meta-Analyses – An Empirical Study
Source: PLoS One. 2016 Dec 7;11(12):e0165889. doi: 10.1371/journal.pone.0165889 (PMC5142775; doi:10.1371/journal.pone.0165889)
Supplement: S1 Appendix — (PDF) [file pone.0165889.s001.pdf]

```
### AB approach

model{
		for(i in 1:nN) {
			p[i]<-phi(mu[t[i]]+ vi[s[i], t[i]])
			y[i]~dbin(p[i], n[i])
		}
		for(j in 1:nS){
			vi[j, 1:nT]~dmnorm(mn[1:nT], T[1:nT,1:nT])
		}

		T[1:nT, 1:nT]<-inverse(invT[ , ])
		for(j in 1:nT){
		    for(k in 1:nT){
			invT[j, k]<-sigma2*pow(rho,step(abs(j-k)-0.5))
		    }
		}

		sigma2<-sigma*sigma
		sigma~dunif(0,5)
		for (j in 1:nT){
			mu[j]~dnorm(0, 0.001)
			probt[j]<-phi(mu[j]/sqrt(1+invT[j, j]))
		}
		rho~dunif(0.01,1)

		for (j in 1:nT){
		    OR[j,j]<-1
			for (k in (j+1):nT){
				OR[k, j] <- probt[k]/(1-probt[k])/probt[j]*(1-probt[j])
				OR[j, k] <- 1/OR[k, j]
			}
		}

		rk<- rank(probt)   # the smaller the better
		for (k in 1:nT) {
			best[k]<-equals(rk[k],1)
		}

#		rk<- nT + 1 - rank(probt)   # the largest the better
#		for (k in 1:nT) {
#			best[k]<-equals(rk[k],1)
#		}
}
```

CB Approach

```
model{
	for(i in 1:NS){
		 w[i,1] <-0
		delta[i,t[i,1]]<-0
		mu[i] ~ dnorm(0,0.1)

		for(k in 1:na[i]){
			r[i,k] ~ dbin(p[i,t[i,k]],n[i,k])
			 logit(p[i,t[i,k]])<-mu[i] + delta[i,t[i,k]]
		 }

		 for(k in 2:na[i]){
			 delta[i,t[i,k]] ~ dnorm(md[i,t[i,k]],taud[i,t[i,k]])
			 md[i,t[i,k]] <-  d[t[i,k]] - d[t[i,1]]  + sw[i,k]
			 taud[i,t[i,k]] <- tau *2*(k-1)/k
			 w[i,k] <- (delta[i,t[i,k]]  - d[t[i,k]] + d[t[i,1]])
			 sw[i,k] <-sum(w[i,1:k-1])/(k-1)
		  }
	}

	d[1]<-0
	 for (k in 2:NT){d[k] ~ dnorm(0,.0001) }

	sd~dunif(0,2)
	tau<-1/pow(sd,2)

# pairwise ORs

	for(j in 1:NT){
		OR[j,j]<-1
		for (k in (j+1):NT){
			lor[k,j] <- d[k] - d[j]
			log(OR[k,j]) <- lor[k,j]
			OR[j, k] <- 1/OR[k, j]
		 }
	  }
}
```

```
### Inconsistency

model{

# likelihood

  for (i in 1:nN) {
    p[i]<-phi(mu[t[i], group[i]] + v[s[i], group[i], t[i]])
    y[i]~dbin (p[i] , n[i])
  }


# random effects

  for (j in 1:nS){
    for(g in 1:n.group) {
      v[j,g,1:nT]~dmnorm(mn[1:nT], T[1:nT, 1:nT])
    }
  }

  T[1:nT, 1:nT]<-inverse(invT[ , ])
  for(j in 1:nT){
    for(k in 1:nT){
      invT[j, k]<-sigma2*pow(rho,step(abs(j-k)-0.5))
    }
  }
  sigma2<-sigma*sigma

# priors

  sigma~dunif(0,5)
  rho~dunif(0.01,1)

  for(k in 1:nT) {
    for(g in 1:n.group) {
      mu[k, g] ~ dnorm(0, 0.001)
    }
  }

# Compute inconsistency between treatments 5 and 1
  dis<-(mu[5,1]-mu[1,1])-(mu[5,3]-mu[1,2])

}
```
